# The Possible Association of Non-Alcoholic Fatty Liver Disease with Acute Cholangitis: A Retrospective Multicenter Cohort Study

**DOI:** 10.3390/life12010035

**Published:** 2021-12-27

**Authors:** Wisam Sbeit, Moeen Sbeit, Itay Kalisky, Lior Katz, Amir Mari, Tawfik Khoury

**Affiliations:** 1 Department of Gastroenterology, Galilee Medical Center, Nahariya 2221006, Israel; wisams@gmc.gov.il (W.S.); moeensbait@gmail.com (M.S.); 2 Faculty of Medicine in the Galilee, Bar-Ilan University, Safed 1320943, Israel; 3 Faculty of Medicine, Institute of Gastroenterology and Hepatology, Hadassah University Medical Center, Hebrew University of Jerusalem, Jerusalem 9103401, Israel; itayk@hadassah.org.il (I.K.); klior@hadassah.org.il (L.K.); 4 Gastroenterology and Endoscopy United, The Nazareth Hospital, EMMS, Nazareth 1613101, Israel; amir.mari@hotmail.com; 5 Faculty of Medicine, Bar-Ilan University, Ramat Gan 5290002, Israel

**Keywords:** cholangitis, NAFLD, severity, CBD, stone

## Abstract

Non-alcoholic fatty liver disease (NAFLD) is increasingly encountered. It is associated with several comorbid diseases. However, its association with infectious biliary diseases is still unknown. **Aims:** We aimed to assess whether NAFLD is a risk factor for the development of acute cholangitis among patients with common bile duct (CBD) stones. **Methods:** We performed a retrospective study, including all patients with a documented diagnosis of CBD stone that had available data on the presence or absence of NAFLD. Descriptive analysis using univariate and multivariate models was used to assess whether an association existed between NAFLD and acute cholangitis. **Results:** We included 811 patients. Of them, 161 patients presented with acute cholangitis, vs. 650 patients who presented with symptomatic CBD stone without cholangitis. NAFLD was significantly more common in the cholangitis group compared to the non-cholangitis group (15.5% vs. 8.3%, *p* = 0.01). In univariate analysis, age (Odds ratio (OR) 1.04, *p* < 0.0001), male gender (OR 1.47, *p* = 0.03), hypertension (OR 1.81, *p* = 0.0008), hyperlipidemia (OR 1.59, *p* = 0.01), and NAFLD (OR 2.04, *p* = 0.006) were significantly associated with acute cholangitis. In multivariate analysis, NAFLD kept its association with acute cholangitis irrespective of age (OR 2.15, *p* = 0.005). **Conclusions:** NALFD showed a significant association with acute cholangitis among patients with a CBD stone. Clinicians should encourage treatment of NAFLD in general, and especially in the setting of gallstone disease.

## 1. Introduction

Symptomatic gallstone diseases are increasingly encountered, accounting for the second-most-common cause of hospital admissions due to gastrointestinal diseases [1,2]. Although only 15% of patients with gallstones become symptomatic [3], the symptomatic disease might lead to life-threatening complications, including biliary pancreatitis and acute cholangitis. Approximately 10–18% of patients who undergo cholecystectomy for gallstones have a concomitant common bile duct (CBD) stone [2]. Acute cholangitis presentation can range from mild, stable disease to severe, fatal disease; therefore, early identification of this gallstone-related biliary complication is essential since prompt antibiotic treatment and early endoscopic drainage minimize morbidity and mortality [4]. The major cause of acute cholangitis is CBD stone; however, the clinical factors that predispose patients with bile duct stone to acute cholangitis are not completely elucidated [5]. Non-alcoholic fatty liver disease (NAFLD) has become the most common cause of chronic liver diseases, constituting a major public health burden [6]. Its prevalence has dramatically increased in western countries, probably related to the tremendous increase in the prevalence of obesity [7]. NAFLD has been associated with several disease states, including breast cancer [8,9], community-acquired pneumonia [10], and colonic hyperplastic polyps [11]. Interestingly, NAFLD was shown to adversely influence the severity and clinical outcome of patients who presented with acute pancreatitis [12]. The aim of the current study is to assess whether NAFLD represents a risk factor for the development of acute cholangitis and whether it affects its severity among patients with CBD stones. 

## 2. Methods

A retrospective, multicenter study was conducted in three hospitals (Galilee Medical Center, EMMS Nazareth hospital, and Hadassah Medical Center). All patients who presented to the hospital due to a documented CBD stone during a 10-year period and who had documented data on the presence or absence of NAFLD were included. The diagnosis of NAFLD was based on the presence of fatty liver on abdominal ultrasound up to 6-months before and after the index admission to the hospital, coupled with the absence of other causes, such as significant alcohol consumption (8 or more drinks per week for women, and 15 or more drinks per week for men) [13], metabolic liver diseases, viral hepatitis and medications (tamoxifen, amiodarone, valproate and steroids). Exclusion criteria included patients who had a confirmed or suspected diagnosis of hepato-pancreato-biliary cancers, patients with primary sclerosing cholangitis, Caroli disease, and patients who had no data regarding their liver status. The study cohort was divided into two groups; group A included patients with clinical presentation of acute cholangitis, and group B included patients with CBD stone-related clinical presentations other than acute cholangitis, such as biliary pancreatitis and biliary colic with abnormal liver enzymes. The groups were then compared for a probable causative effect of NAFLD presence on cholangitis or its severity. All patients’ files were reviewed, and demographic data, medical history, gallbladder status, presence of gallstones, and NAFLD were extracted. Moreover, data regarding acute cholangitis severity were extracted. The study was approved by the local ethics committee of all three institutions. Written informed consent was waived due to the retrospective non-interventional design of our study.

### 2.1. Study Definitions

#### 2.1.1. Acute Cholangitis

Acute cholangitis in our study was defined according to Tokyo guidelines, characterized by the presence of fever of >38 °C, or shivering and jaundice, coupled with the presence of elevated inflammatory markers, abnormal liver enzymes, and evidence of a common bile duct stone or dilated CBD on imaging. Severe cholangitis was diagnosed when there was associated organ dysfunction, including hemodynamic instability, requiring inotropic vasopressors support, respiratory failure, renal failure, hepatic failure, neurological impairment, and coagulopathy; moderate cholangitis was defined by the presence of two out of five parameters, including: white blood count > 12 thousand or < 4 thousand, fever ≥ 39 degree Celsius, age > 75 years, total bilirubin > 5 mg/dL, or hypoalbuminemia; and mild cholangitis was diagnosed when none of the above criteria were met [14].

#### 2.1.2. Study Aims

The main aim of our study was to assess the association of the presence of NAFLD with the development of acute cholangitis in patients with a CBD stone. The secondary aim was to assess whether the presence of NAFLD was associated with a higher cholangitis severity. Identifying a positive correlation between NAFLD and acute cholangitis development among patients with gallstone disease is of paramount importance since NAFLD is a reversible and treatable disease by lifestyle modification and weight loss, which might reduce the risk of acute cholangitis development.

### 2.2. Statistical Analysis

Continuous variables were presented as arithmetic means (±standard deviation (SD)) or a range, while categorial variables were presented as frequencies (percentages). Univariate analysis was used to assess the association between the presence of NAFLD and the development of acute cholangitis by reporting the odds ratio (OR) and confidence interval (CI). To eliminate the effect of cofounders, multivariate logistic regression analysis by performing a backward selection model was used to explore the final effect of NAFLD on acute cholangitis. Statistical significance was set at a *p*-value less than 5%. Statistical analysis was performed with commercial software by an experienced statistician using statistical analysis software (SAS vs. 9.4 Copyright (c) 2016 by SAS Institute Inc., Cary, NC, USA).

## 3. Results

**Figure 1:**
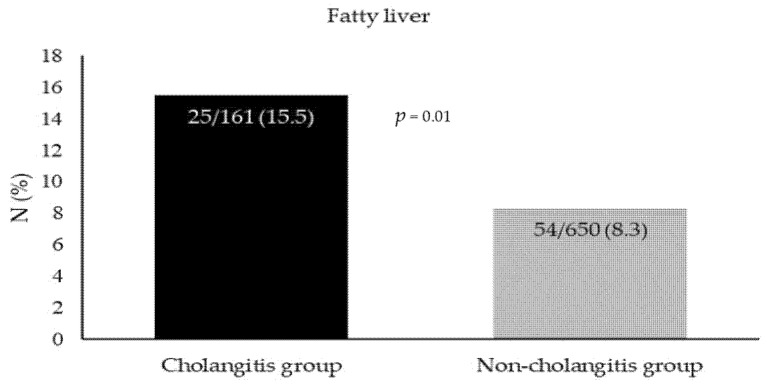
The distribution of patients with and without cholangitis.

Overall, our cohort consisted of 811 patients. Of them, 161 patients presented with acute cholangitis secondary to a CBD stone (cholangitis group), vs. 650 patients who presented with a symptomatic CBD stone without cholangitis (non-cholangitis group). The mean age in the cholangitis group was higher compared to the non-cholangitis group (74.5 ± 15.6 vs. 61.6 ± 20.9). There was no difference in the gender distribution between the two groups. Similarly, there was no difference in medical history between the two groups. Of note, NAFLD was significantly more common in the cholangitis group compared to the non-cholangitis group (15.5% vs. 8.3%, *p* = 0.01) (Figure 1). Notably, there was no difference in the use of Dipeptyl Peptidase-IV Inhibitors (DPP-4) inhibitor medication (1.9% in the cholangitis group vs. 0.6% in the non-cholangitis group, *p* = 0.14). Table 1 demonstrates the study baseline characteristics.

### 3.1. Laboratory Findings among the Study Cohort

The hemoglobin level was 12.3 ± 1.9 g/dL in the cholangitis group, compared to 12.5 ± 1.9 g/dL in the non-cholangitis group (*p* = 0.18). Similarly, there was no difference in creatinine level, alanine aminotransferase (ALT), amylase, and gamma-glutamyl transferase (GGT) (1.1 ± 0.86 mg/dL, 182.5 ± 212.6 IU/L, 147 ± 311.3 IU/L, and 496.7 ± 404.6 IU/L) in the cholangitis group, compared to 1.1 ± 2.4 mg/dL, 177 ± 197 IU/L, 181.1 ± 505 IU/L, and 453 ± 389 IU/L in the non-cholangitis group (*p* = 0.45, *p* = 0.39, *p* = 0.25 and *p* = 0.13), respectively. On the other hand, the neutrophil count (15.8 ± 22.1 10^9^/L vs. 10 ± 17.4 10^9^/L, *p* = 0.0004), aspartate transferase (AST) (149.7 ± 191.5 IU/L vs. 112.8 ± 142 IU/L, *p* = 0.006), alkaline phosphatase (ALP) (289 ± 206.9 IU/L vs. 230.2 ± 185.4 IU/L, *p* = 0.0005), total bilirubin (7.3 ± 7 mg/dL vs. 3 ± 6.9 mg/dL, *p* = 0.01), and C-reactive protein (CRP) (75.9 ± 86 mg/L vs. 43.3 ± 66.1 mg/L, *p* < 0.0001) were significantly higher in the cholangitis group, compared to the non-cholangitis group, respectively.

### 3.2. Univariate and Multivariate Analysis of Factors Correlated with the Development of Acute Cholangitis

In univariate analysis, we found that two non-modifiable parameters were associated with acute cholangitis, including age (OR 1.04, 95% CI 1.03–1.05, *p* < 0.0001) and male gender (OR 1.47, 95% CI 1.04–2.08, *p* = 0.03). Moreover, three modifiable parameters were associated with acute cholangitis, including hypertension (OR 1.81, 95% CI 1.28–2.57, *p* = 0.0008), hyperlipidemia (OR 1.59, 95% CI 1.11–2.29, *p* = 0.01), and NAFLD (OR 2.04, 95% CI 1.23–3.39, *p* = 0.006). In multivariate logistic regression analysis, NAFLD and age kept their association with acute cholangitis (OR 2.15, CI 1.25–3.72, *p* = 0.005 and OR 1.037, CI 1.02–1.05, *p* < 0.0001), respectively (Table 2).

### 3.3. Association of NAFLD with Severity of Acute Cholangitis

**Figure 2:**
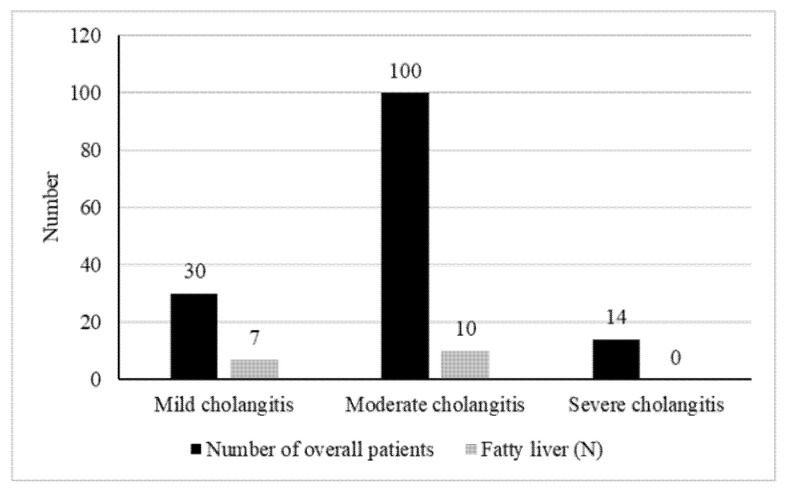
The prevalence of NAFLD among the different cholangitis severity groups.

Overall, cholangitis severity was available in 144 patients. Among them, 30 patients had mild disease, 100 had moderate disease, and 14 patients had severe disease (Figure 2). Among the mild and moderate cholangitis groups, 7 patients (23.3%) and 10 patients (10%) had NAFLD, while none of the patients had NAFLD in the severe cholangitis group. Of note, NAFLD was not associated with either moderate or severe cholangitis compared to mild disease (OR 0.37, *p* = 0.06) and (OR 0.11, *p* = 0.13), respectively. Moreover, NAFLD had no effect on severe vs. moderate cholangitis (OR 0.29, *p* = 0.41). Table 3 demonstrates the statistical significance of the associations of NAFLD with the different cholangitis severity groups.

## 4. Discussion

The pandemic of obesity is expanding worldwide to an overall prevalence of 13% among the adult population. The World Health Organization (WHO) has estimated in its revised publication from June 2021 that in 2016, at least 1.9 billion adults were overweight, and over 650 million among them were obese, with major consequences on public health. In parallel with obesity, NAFLD has climbed to become the leading cause of chronic liver disease worldwide, with a prevalence of 33% [15]. Apart from its association with metabolic syndrome, NAFLD has emerged as a risk factor for several extrahepatic diseases, including cardiovascular diseases and extrahepatic cancers. It is suggested that NAFLD induces a low-grade inflammatory state in proportion with liver disease severity through the systemic release of inflammatory cytokines, procoagulant factors, and oxidative stress [16]. This association is postulated to be secondary to the interaction between the gut and liver named the Gut–Liver Axis. The gut microbiome includes all microorganisms in the gastrointestinal tract. The imbalance of the bacteria found in the gut is known as dysbiosis. Dysbiosis has been linked to the development of various metabolic conditions, including obesity and NAFLD [17,18]. Moreover, bacterial dysbiosis may lead to increased inflammation, lipotoxicity, choline, and bile acid metabolism disorders, which have all been thought important to the development of cholangitis. Previous studies have found high numbers of Fusobacteria and reduced richness of Oscillospira and Ruminococcus in NAFLD patients [19]. Additionally, studies have shown a relationship between bile acids and the gut microbiome in NAFLD pathogenesis, particularly via bile acids’ role in lipid metabolism [20]. Moreover, a previous study showed a significant association between the primary bacteremia of the presumed gastrointestinal origin of NAFLD [21], further supporting the role of the Gut–Liver Axis in developing acute infectious processes, especially acute cholangitis.

In our study, we could clearly show a close relationship between NAFLD and the development of acute cholangitis in patients with a CBD stone in multivariate analysis (odds ratio 2.15, 95% confidence interval 1.25–3.72, *p*-value 0.005). NAFLD and gallstone disease share several risk factors, including ethnicity, age, and metabolic syndrome [22,23]. A systematic review and meta-analysis of an overall 79,629 patients reported a significant association between gallstone disease and NAFLD [24]. It could be assumed that the prevalence of CBD stone is supposed to increase with increasing evidence of gallbladder stones. Probably, the low-grade inflammatory state induced by NAFLD predisposes these patients to cholangitis, as was shown in our study. As NAFLD is a modifiable risk factor, this life-threatening association of cholangitis merits concern, as treating NAFLD could probably reduce the prevalence of cholangitis in these patients. Notably, a recent systematic review with the meta-analysis by Váncsa et al. reached the conclusion that NAFLD worsens the outcome of acute pancreatitis and suggested incorporating it into prognostic scores of acute pancreatitis outcome [25]. However, in our study, NAFLD was not associated with the severity of cholangitis. An association of cholangitis in patients with CBD stone was also demonstrated with age. Advanced age has already been reported by previous studies to be a risk factor for acute cholangitis [26,27]. It could be explained by several age-related factors, including multiple chronic diseases, reduced mobility, age-related decrease in host defense, lower physiologic reserve, and multiple medications being used [28]. To summarize, NAFLD and gallstone disease are commonly encountered diseases worldwide with increasing incidence, which lead to the development of several comorbid and life-threatening diseases, including cirrhosis, hepatocellular carcinoma, and acute cholangitis. Therefore, as we found a significant association between NAFLD and acute cholangitis in our study, it is of paramount importance to identify those patients, as the early treatment of NAFLD by implementing lifestyle modification, weight loss, and NAFLD-targeted medication might minimize morbidity and mortality that can complicate gallstone disease.

The limitations of our study are its retrospective nature of data collection and the fact that we did not discriminate between non-alcoholic steatohepatitis (NASH) and NAFLD patients, as the systemic inflammatory state accompanying NAFLD increases with the advance of the liver disease to NASH. Another important limitation is the missing data on cholangitis severity in 17 patients, as these missing data might underestimate our results. Therefore, further prospective studies are warranted to exactly assess the effect of NAFLD on acute cholangitis severity. An additional limitation is the lack of data on weight variation, BMI, and obesity prevalence.

In conclusion, in our study, we showed a close relationship between NAFLD and acute cholangitis in patients with CBD stone. The rising prevalence of NAFLD that accompanies the rise in obesity worldwide could represent a game-changer in the etiopathogenesis of the life-threatening acute cholangitis, an association that needs special attention, as early recognition and treatment could be lifesaving. Additionally, it represents another important reason to encourage patients to lose weight and thus improve their NAFLD and prevent acute cholangitis.

## Figures and Tables

**Table 1:** Demographics and baseline characteristics.

	Cholangitis Group	Non-Cholangitis Group	*p*-Value
Number of patients	161	650	-
Age, mean ± SD (years)	74.5 ± 20.6	61.6 ± 20.9	0.001
Male/Female, *N* (%)	80 (49.7)/81 (50.3)	261 (40.1)/389 (59.9)	0.02
Chronic liver disease, *N* (%)	9 (5.6)	29 (4.5)	0.54
Alcohol, *N* (%)	2 (1.2)	6 (0.9)	0.71
Diabetes mellitus, *N* (%)	64 (39.7)	211 (32.5)	0.08
Hyperlipidemia, *N* (%)	58 (36)	170 (26.1)	0.01
Hypertension, *N* (%)	93 (57.8)	279 (42.9)	0.007
Chronic renal failure, *N* (%)	8 (5)	32 (4.9)	0.98
Single CBD stone, *N* (%)	20 (12.4)	62 (9.5)	0.28
Multiple CBD stones, *N* (%)	135 (83.8)	554 (85.2)	0.66
Gallbladder stones, *N* (%)	111 (68.9)	474 (72.9)	0.31
Dipeptyl Peptidase-IV Inhibitors (DPP-4) inhibitor, *N* (%)	3 (1.9)	4 (0.6)	0.12
glucagon-like peptide 1 (GLP-1) analogues, *N* (%)	0	0	-

Mann–Whitney and Fisher’s Exact Probability tests were used where appropriate.

**Table 2:** Univariate and multivariate analysis of parameters associated with acute cholangitis.

Univariate Analysis			
	Odds Ratio	95% CI	*p*-Value
Male gender	1.47	1.04–2.08	0.03
Age	1.04	1.03–1.05	<0.0001
Alcohol	1.55	0.33–7.35	0.58
Diabetes mellitus	1.38	0.96–1.96	0.08
Chronic liver disease	1.31	0.61–2.80	0.49
Gallbladder stones	0.96	0.64–1.43	0.82
Chronic renal failure	1.05	0.48–2.31	0.89
Hyperlipidemia	1.59	1.11–2.29	0.01
Hypertension	1.81	1.28–2.57	0.0008
Stone number	1.34	0.78–2.29	0.28
NAFLD	2.04	1.23–3.39	0.006
**Multivariate Analysis**			
Age	1.037	1.02–1.05	<0.0001
NAFLD	2.15	1.25–3.72	0.005

Logistic regression with a backward selection model was used, calculated by Fisher’s Exact Probability test.

**Table 3:** Correlations between cholangitis severity grades and NAFLD presence.

	Odds Ratio	95% CI	*p*-Value
Moderate vs. mild cholangitis	0.37	0.13–1.06	0.06
Severe vs. mild cholangitis	0.11	0.01–2.04	0.13
Severe vs. moderate cholangitis	0.29	0.02–5.35	0.41

Fisher’s Exact Probability test was used to assess correlations.

## Data Availability

The data are available with the corresponding author at the Gastroenterology department at Galilee Medical Center and it will be available upon reasonable request.

## Abbreviations

NAFLD: Non-alcoholic fatty liver disease; CBD: common bile duct; OR: Odds ratio; ESGE: European Society of Gastrointestinal Endoscopy; SD: standard deviation; CI: confidence interval; WHO: world health organization; NASH: non-alcoholic steatohepatitis.
